# Characterization of the Antiproliferative and Antimetastatic Properties of *Centrapalus pauciflorus* Meroterpenoid Centrapalus Coumarin F

**DOI:** 10.3390/ijms26104489

**Published:** 2025-05-08

**Authors:** Hazhmat Ali, Shelan Rasool, Muhammad Bello Saidu, Péter Germán, Gábor J. Szebeni, Enikő Szabó, Dóra Rédei, Judit Hohmann, István Zupkó

**Affiliations:** 1 Institute of Pharmacodynamics and Biopharmacy, University of Szeged, 6720 Szeged, Hungary; ali.hazhmat@gmail.com (H.A.); shelan.rasool@uod.ac (S.R.); petergerman1974@gmail.com (P.G.); 2 College of Medicine, University of Duhok, Duhok 42001, Kurdistan Region, Iraq; 3 Institute of Pharmacognosy, University of Szeged, 6720 Szeged, Hungary; mbellosaidu11@gmail.com (M.B.S.); redei.dora.judit@szte.hu (D.R.); hohmann.judit@szte.hu (J.H.); 4 Laboratory of Functional Genomics, Core Facility, HUN-REN Biological Research Centre, 6726 Szeged, Hungary; szebenigabi@gmail.com (G.J.S.); epermarion@gmail.com (E.S.); 5 Department of Internal Medicine, Hematology Centre, University of Szeged, 6720 Szeged, Hungary; 6 HUN-REN-USZ Biologically Active Natural Products Research Group, University of Szeged, 6720 Szeged, Hungary; 7 Interdisciplinary Centre of Natural Products, University of Szeged, 6720 Szeged, Hungary

**Keywords:** antiproliferative, antimetastatic, centrapalus coumarin F, *Centrapalus pauciflorus*

## Abstract

The current study examined the in vitro antineoplastic potentials of centrapalus coumarin F (CCF) obtained from aerial parts of *Centrapalus pauciflorus* (Willd.) H.Rob. (Asteraceae). Cytotoxic activity was tested against a panel of human adherent cancer cell lines, including breast, cervical, and oropharyngeal cancer and glioblastoma. Cell cycle analyses using flow cytometry and Hoechst 33258-propidium iodide (HOPI) fluorescent double staining were used to describe the proapoptotic property of CCF. Wound healing assessment and the Boyden chamber assay were performed to characterize the antimetastatic action of the compound. The firefly luciferase assay was applied to clarify the action of CCF on estrogenic receptors. CCF demonstrated remarkable selective growth inhibition against the HPV-18-positive human cervical cancer cell line HeLa (IC_50_ = 2.28 µM). The compound elicited crucial markers of apoptosis, inhibited the migration and invasion capacity of HeLa cells, and demonstrated an antiestrogenic property. Our current data indicate that the meroterpenoid scaffold presented here displays remarkable antiproliferative and antimetastatic effects on HeLa cells and can be considered a valuable model for designing further analogs targeting cervical carcinoma.

## 1. Introduction

Cancer is a diverse group of diseases that occur in various tissues and organs of the body due to a series of genetic and environmental alterations [1]. Its mortality rate consistently places the group as the second cause of death after cardiovascular diseases [2]. In addition to surgery and radiation, chemotherapy has been considered the golden option for the clinical management of various types of cancers; however, high toxicity and multidrug resistance remain the main obstacles [3,4,5]. Due to this, greater attention is being paid to the development of other classes of therapeutic anticancer agents that exert exceptional anticancer effects with low toxicity, as well as acting through different mechanisms of actions, such as alkylating agents, antimetabolites, hormones, antagonists, and even natural products [6,7].

Natural compounds represent a vital source of anticancer agents in drug discovery. Approximately 80% of anticancer agents are estimated to be derived from natural products [8]. Meroterpenoids are terpenoid-containing natural hybrid products with versatile structural architectures and impressive pharmacological properties. The non-terpenoid moiety of the molecules may have a polyketide, shikimate, or alkaloid origin [9]. Monoterpenoid-coupled coumarins represent a minor group of meroterpenoids that are rare compounds in plants that usually occur together with structurally related monoterpenoid chromones in the genera of the family Asteraceae [10,11]. The biological activity of coumarin–monoterpene adducts has currently barely been explored; however, simple coumarins are abundantly found in many dietary sources, such as in fruits (e.g., *Aegle marmeleos* L., Rutaceae), seeds (e.g., *Calophyllum inophyllum* L., Calophyllaceae), roots (e.g., *Ferulago campestris* (Besser) Grec., Apiaceae), and leaves (e.g., *Murraya paniculata* L., Rutaceae) [12]. With regard to anticancer potential, coumarin and its derivatives have been shown to possess anticancer activity against various cancers, mainly prostate, kidney, breast, lung, and colon cancer, leukemia, and malignant melanoma [13,14,15]. Coumarin derivatives exert their potential effects by easily interacting with various receptors and enzymes such as kinase, aromatase, sulfatase, and monocarboxylate transporters [16]. Scopoletin, one of the most abundant coumarins, has been found to inhibit the growth of cervical cancer cells due to the induction of apoptotic cell death and cell cycle arrest and inhibit cell invasion [17].

A previous study conducted by our group investigated the antiproliferative activities of thirteen undescribed monoterpene-fused 5-methylcoumarins together with seven known compounds isolated from the aerial parts of *Centrapalus pauciflorus* (Willd.) H.Rob. (Asteraceae) [18]. They were tested against a panel of human gynecological cancer cell lines, including breast (MCF-7 and MDA-MB-231), cervical (HeLa and SiHa), and ovary (A2780) cancer cells, using the MTT assay. One of the compounds tested, Centrapalus coumarin F (CCF), exerted substantial growth inhibition against the HPV-18-positive human cervical cancer cell line (HeLa); its calculated IC_50_ value was 2.28 µM [18]. Based on the above literature overview, we assume that the coumarin scaffold may be a valuable model for designing more analogs targeting carcinomas. The present study aimed to explore the pharmacological properties of CCF, including its antiproliferative and antimetastatic actions on HeLa cells.

## 2. Results

### 2.1. Antiproliferative Properties of CCF

**Figure 1:**
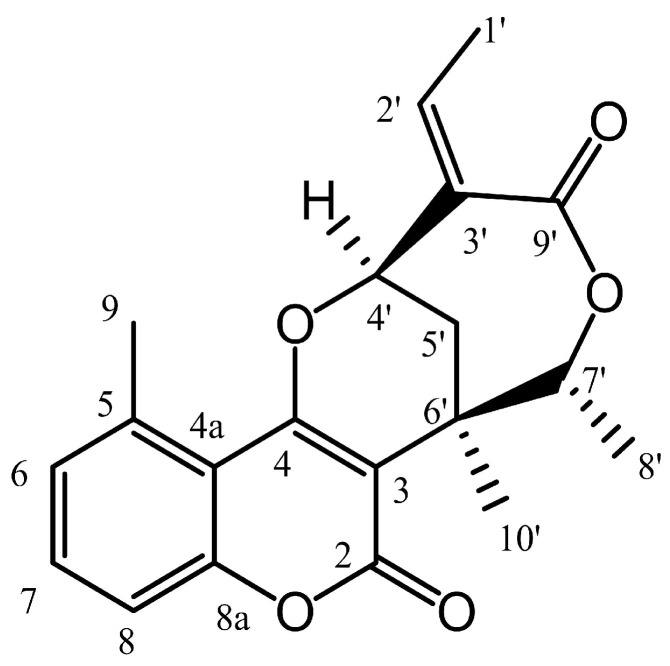
Chemical structure of centrapalus coumarin F (CCF).

The in vitro antiproliferative activities of the investigated natural product (Figure 1) were previously tested against a panel of human gynecological cancer cell lines [18]. Since the compound exhibited considerable effects, especially against HPV-18-positive cervical carcinoma cells (HeLa, IC_50_ = 2.28 μM), further human cancer cell lines were employed in its investigation (Table 1).

These included breast (T47D) and cervical (C33A) cell lines, as well as additional cells isolated from non-gynecological malignancies: two oropharyngeal (UPCI-SCC-131, UPCI-SCC-154) cell lines and a glioblastoma (U-87) cell line. The test compound showed moderate inhibition against cervical (C33A) and breast cancer (T47D) cell lines, generating IC_50_ values of 15.69 and 25.55 μM, respectively. The test compound moderately inhibited the proliferation of the glioblastoma cells, and the two oropharyngeal cell lines were even less sensitive. Therefore, HeLa cells remained the most sensitive cell line, in which CCF elicited the lowest calculated IC_50_ value. Non-cancerous mouse fibroblast cells (NIH/3T3) were also used to obtain results regarding CCF tumor selectivity. The natural product tested in our study moderately inhibited the proliferation of fibroblasts; a more than ten-fold higher IC_50_ value was calculated compared to that of HeLa cells. Based on these results, our additional experiments were carried out using HPV-18-positive cervical cancer cells.

### 2.2. CCF Induced G0/G1 Cell Cycle Arrest

**Figure 2:**
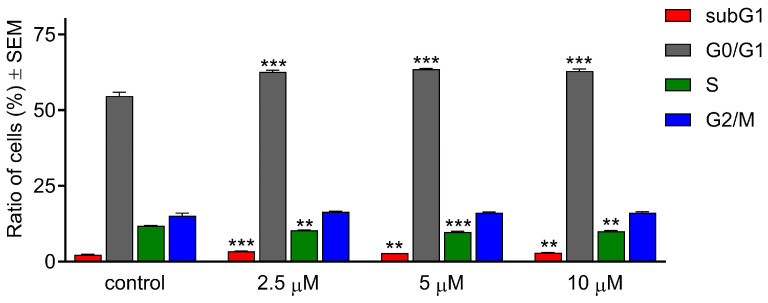
Cell cycle distribution of HeLa cells after 24 h of treatment with CCF. Results from three independent experiments carried out in triplicate. ** and *** indicate significance at *p* < 0.01 and *p* < 0.001, respectively.

Cell cycle analysis was performed using flow cytometry to approach the mechanism of the antiproliferative action of CFF. HeLa cells were treated with selected concentrations (2.5, 5, and 10 µM) and incubated for 24 h (Figure 2).

The cell cycle distribution showed a statistically significant increase in the G0/G1 cell ratio at the expense of the S phase cell population, indicating a blockade in the G0/G1 transition. A noticeable elevation was also detected in the subG1 population in the treated groups compared to the control. However, no notable changes were observed in the population of G2/M cells.

### 2.3. CCF Induced Morphological Changes

**Figure 3:**
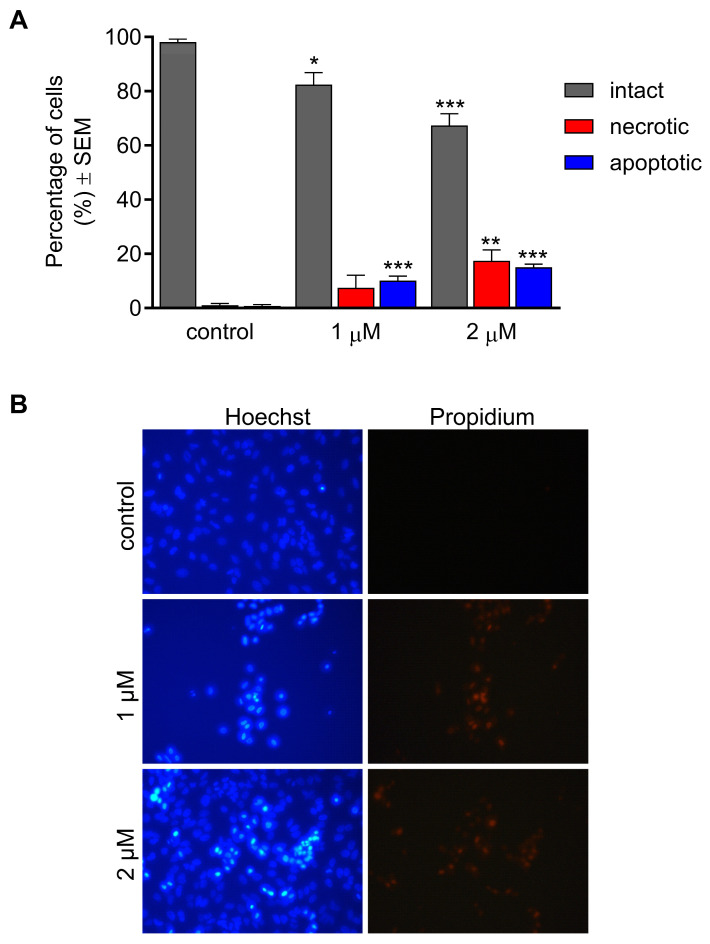
Proportion of intact, apoptotic, and necrotic HeLa cells after 24 h of CCF treatment (**A**) and representative images, magnification: 40× (**B**). Data from two independent experiments performed in triplicate. *, **, and *** indicate significance at *p* < 0.05, *p* < 0.01, and *p* < 0.001, respectively.

Hoechst 33258 and propidium iodide (HOPI) fluorescence double staining was performed to investigate the ability of CCF to induce morphological changes. HeLa cells were treated with two concentrations (1 and 2 µM) and incubated for 24 h. A significant concentration-dependent decrease in the intact cell population was observed after exposure (Figure 3).

Cell populations that emit blue fluorescence from accumulated Hoechst 33258 were significantly elevated at both applied concentrations, indicating treatment-related nuclear condensation. Red fluorescence is a consequence of propidium-positive nuclear staining, which is regarded as a marker of severe cell membrane damage. This damage can be attributed to the development of necrotic cell death. The portion of necrotic cells with damaged membranes was significantly higher after exposure to 2 μM CCF.

### 2.4. Effect of CCF on Cancer Cell Migration

**Figure 4:**
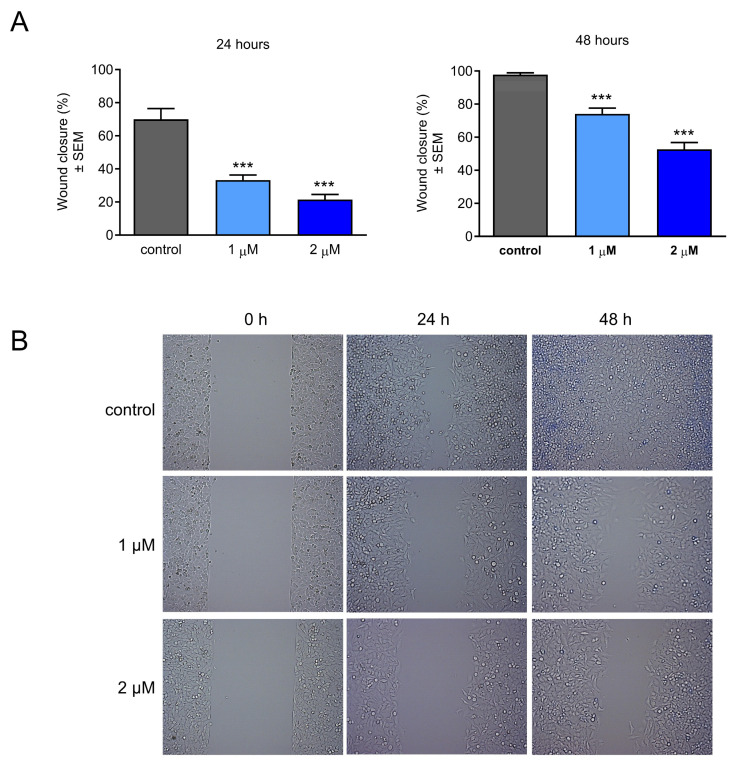
Antimigratory action of CCF on HeLa cells after 24 and 48 h of treatment (**A**) and representative pictures (**B**). Results from three independent experiments carried out in triplicate. *** indicates significance at *p* < 0.001.

Besides the direct antiproliferative action of CCF, its antimetastatic properties could also be perspectivistic. Therefore, a wound-healing assay was performed to determine the effects of the test compound on cell migration. For this purpose, HeLa cells were treated with two different concentrations (1 and 2 µM) and incubated for 24 or 48 h. The results showed significant concentration-dependent antimigratory actions after both treatment durations, even at the lower concentration (Figure 4).

### 2.5. Effect of CCF on Cancer Cell Invasion

**Figure 5:**
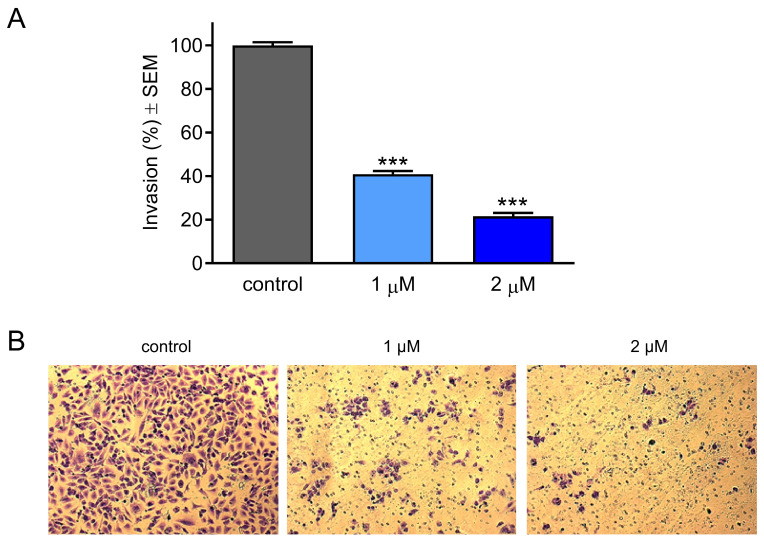
Effect of CCF on the invasive capacity of HeLa cells after 24 h of treatment (**A**) and representative images, magnification: 10× (**B**). Results from three independent experiments carried out in triplicate. *** indicates significance at *p* < 0.001.

A Boyden chamber assay was performed to explore the anti-invasive capacity of the test compound. HeLa cells were treated with 1 or 2 µM of CCF and incubated for 24 h under previously described culture conditions. Based on the results obtained, CCF exerted a statistically significant concentration-dependent decrease in the percentage of invaded cells compared to the untreated control condition (Figure 5).

### 2.6. CCF Exerted an Antiestrogenic Effect

**Figure 6:**
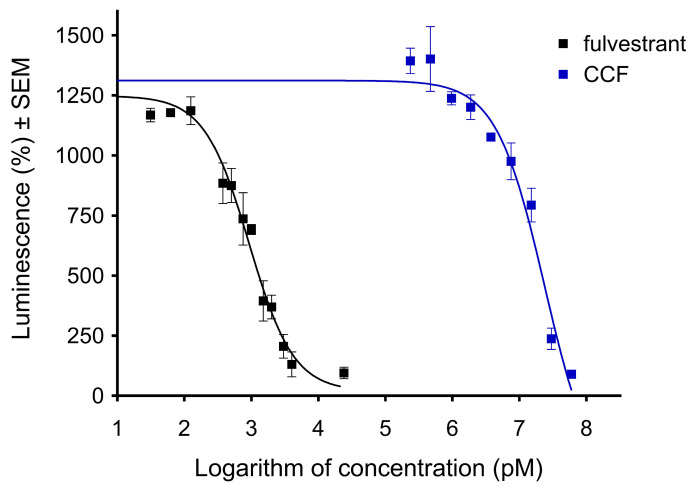
The antiestrogenic effect of CCF determined by employing a luciferase reporter gene assay. Results from two independent experiments carried out in triplicate.

A cancer cell line transfected with an estrogen-responsive firefly luciferase reporter gene (T47D-KBluc) was applied to characterize the action of CCF on the estrogen receptor. The receptors of the transfected cells were stimulated with 40 pM 17β-estradiol, while fulvestrant, a selective estrogen receptor degrader, was used as a reference agent. According to the results, CCF can inhibit the action of 17β-estradiol, though this effect requires a substantially higher concentration than in the case of fulvestrant (Figure 6). The calculated IC_50_ values for the reference agent and the CCF are 0.922 nM and 23.97 μM, respectively. Therefore, CCF exhibits antiestrogenic properties at concentrations approximately ten times higher than its antiproliferative and antimetastatic activities against HeLa cells.

## 3. Discussion

Data from the previous literature demonstrated that naturally derived coumarins can provide a valuable base for designing more potent compounds that induce anticancer activities [19,20,21,22,23]. Moreover, coumarins are widely applied in hybridization approaches to generate novel anticancer drug candidates. They have been coupled to a variety of heterocyclic rings, as well as other natural products, including chalcones, artemisinin, and different monoterpenes [24,25]. Our present study aimed to investigate the antiproliferative and antimetastatic potentials of the coumarin–monoterpene adduct CCF and to approach its mechanism of antiproliferative action. Our group recently reported CCF as a previously unknown natural product, and its antiproliferative properties were tested against a panel of human gynecological cancer cell lines [18]. Since it proved to be a promising compound, we attempted to further investigate its antiproliferative properties by testing it against further malignant cell lines with different receptor expression and phenotype identification, including breast (T47D), cervical (C33A), and oropharyngeal (UPCI-SCC-131 and UPCI-SCC-154) cancer cells and glioblastoma (U-87). Among these newly included cell lines, the test compound moderately inhibited only papillomavirus-negative cervical cancer cells (C33A). Consistently, other cell lines did not show any notable activity. The cancer selectivity of the molecule was additionally tested by determining its cell growth-inhibitory action against non-cancerous NIH/3T3 cells. A viability assay against mouse fibroblasts cannot be considered to provide a fully reliable description of the tolerability of an anticancer drug candidate. However, the more than tenfold difference in IC_50_ values can be regarded as promising in terms of general toxicity.

The HPV-18-positive HeLa cell line was selected for the further evaluation of CCF based on previously confirmed outstanding growth inhibition. Since identifying the mechanism of action of anticancer agents is essential in pharmacodynamic research, we investigated the influence of CCF on cell cycle analysis using flow cytometry. Since the assayed events, including cell cycle disturbance or membrane damage, are expected within a doubling time, shorter CCF exposures were applied.

The test compound induced the arrest of the cell cycle in G0/G1 at the expense of the S cell population. These alterations in cell cycle behavior are consistent with previous studies, indicating that the G0/G1 cell cycle arrest is closely associated with the antiproliferative activity of the tested substances, including natural products [26,27,28]. This was further evidenced by measuring cell cycle-dependent proteins, which showed the down-regulation of cyclin-dependent kinase 4 (CDK4) and increased cyclin D1 [26,28]. Based on this concept, we assume that these changes in the cell cycle distribution induced by CCF may reflect its potent antiproliferative activity. Programmed cell death modalities, including apoptosis, constitute a crucial physiological process that restricts the expansion of cell proliferation, either to maintain homeostasis or to remove undesirable cells with sustained DNA damage [29]. The molecular mechanism of apoptosis is well understood and requires the extensive activation of caspase proteases through extrinsic or intrinsic pathways [30,31]. In cancer research, antineoplastic agents that are capable of induced apoptosis are currently receiving significant attention because one of the hallmarks by which cancer cells escape immune surveillance is through the evasion of apoptosis [32]. In that context and based on the noticeable elevation in the subG1 cell population in the cell cycle, we evaluated the morphological changes induced by our test compound using HOPI fluorescent double staining. The CCF-induced morphological changes were characterized by a concentration-dependent reduction in the percentage of intact cells with a significant elevation in the populations of necrotic and apoptotic cells. Based on these findings, it can be concluded that CCF has the ability to induce morphological changes represented by necrosis and apoptosis and may possess proapoptotic potential. However, identifying the precise mechanism of apoptotic induction still requires further investigation. Our data are supported by previous studies confirming the proapoptotic effects of various natural and semisynthetic coumarins that induce apoptosis in different cancer cell lines, including acute myeloid leukemia, breast and colorectal, hepatic, and pancreatic cancers [33,34,35,36].

Metastatic potency is considered one of the hallmarks of cancer. It involves disseminating cells from primary tumor sites to distant organs such as the lungs, liver, and brain [37]. This process requires a series of cellular events including migration, invasion, dissemination, and escape from immune surveillance. Due to the complexity of the tumor microenvironment, the molecular characterization of metastasis is not well understood [38,39]. Because the generation of secondary tumors involves a complex interplay between genetic and epigenetic factors, numerous investigations have aimed to develop therapeutic agents that target metastasis, thus decreasing the mortality rate and improving the efficacy of therapeutic antineoplastic agents [40,41,42]. Since migration and invasion are critical factors in the metastatic process, our objective was to investigate the antimetastatic effects of CCF using wound healing and Boyden chamber assays, respectively. On the basis of the obtained data, the migration and invasion of HeLa cells were substantially inhibited by our test compound in a concentration-dependent manner. Furthermore, these actions were elicited considerably below its antiproliferative IC_50_ value, indicating that they could be independent of the inhibition of cell growth. It can be concluded that CCF exhibits a remarkable antimetastatic action against HeLa cells.

Estrogens exert various physiological functions, from regulating reproductive functions to modulating bone density and cholesterol mobilization [43]. However, chronic exposure to exogenous estrogens can increase the risk of developing various types of gynecological cancer, mainly breast, ovarian, and cervical carcinomas [44,45,46]. Therefore, the estrogen-antagonistic behavior of a directly acting anticancer drug candidate can contribute to its overall pharmacological relevance. This multiple mechanism can be exploited for the treatment of hormone-dependent malignancies. Since coumarins are known for their antiestrogenic action, it was rational to investigate CCF for this activity [47]. The estrogenic behavior of CCF was characterized by testing it using the genetically modified T47D cell line expressing an estrogen-responsive element (T47D-KBluc). CCF displayed moderate antagonistic effects on the applied system, decreasing the action of 17β-estradiol. Our findings are consistent with previous reports on the estrogenic profiles of various natural and semisynthetic coumarins. It has been highlighted that, in addition to their potent cytotoxic effects against breast cancer cell lines, coumarins of different chemical backgrounds have exhibited strong estrogen receptor alpha antagonistic effects, making them suitable candidates for developing anticancer agents with multiple sites of action [47,48,49,50].

## 4. Materials and Methods

### 4.1. Test Compound

Centrapalus coumarin F (CCF) represents a naturally derived coumarin–monoterpene-based meroterpenoid isolated from the aerial parts of *Centrapalus pauciflorus* (Asteraceae). Its isolation process and the structure elucidation were previously described [18].

### 4.2. Tumor Cell Lines and Culture

All cell lines were purchased from the European Collection of Cell Cultures (ECCAC, Salisbury, UK) except for C33A (American Tissue Culture Collection, Manassas, VA, USA) and oropharyngeal cell lines (German Collection of Microorganisms and Cell Cultures GmbH, Braunschweig, Germany). In this study, a panel of human cancer cell lines was utilized, including breast (T47D), cervical (C33A and HeLa), and oropharyngeal (UPCI-SCC-131 and UPCI-SCC-154) cancer cells, as well as glioblastoma (U-87). The cancer selectivity of CCF was tested on NIH/3T3 non-cancerous fibroblasts isolated from mouse embryos. Culture media and supplements were purchased from Capricorn Scientific Ltd. (Ebsdorfergrund, Germany), while other chemicals used in the experiments, unless indicated otherwise, were obtained from Merck Life Science Ltd. (Budapest, Hungary). All cells (except for glioblastoma) were maintained in Eagle’s minimum essential medium (EMEM) supplemented with 10% fetal bovine serum, 1% non-essential amino acids, and 1% antibiotic–antimycotic mixture at 37 °C in humidified air containing 5% CO_2_. Regarding glioblastoma, cells were kept in a special medium containing the above supplements in addition to a 1% sodium-pyruvate solution and 1% L-glutamine.

### 4.3. Antiproliferative Assay

The cytotoxic effect of CCF against cancer cell lines was tested using the standard MTT assay. The experiments were repeated on a non-cancerous mouse fibroblast cell line (NIH/3T3) to determine tumor selectivity. Cells were seeded in 96-well plates at a density of 5000 cells/well, except for C33A, seeded at 10,000 cells/well, and incubated overnight. The cells were then treated with the desired concentrations of CCF and incubated for 72 h under standard culture conditions. Following the incubation period, 20 µL of MTT solution [3-(4,5-dimethylthiazol-2-yl)-2,5-diphenyltetrazolium bromide] was added to each well and incubated for 4 h. The supernatants were gently removed, and the precipitated blue formazan crystals were dissolved by adding 100 µL of dimethylsulfoxide (DMSO, Reanal, Budapest, Hungary) to each well, followed by shaking for 60 min. Finally, the absorbance values were recorded by a microplate reader (SPECTROstar Nano, BMG Labtech GmbH, Offenburg, Germany) at 545 nm. In the first step, the action of the compound was assayed at two concentrations (10 and 30 μM), and if a cell growth inhibition of greater than 50% was obtained at 30 μM, the assay was repeated with a set of dilutions. Sigmoidal concentration–response curves were fitted, and IC_50_ values were calculated using GraphPad Prism 5.01 software (GraphPad Software, San Diego, CA, USA). Untreated cells were used as a control condition. All experiments were repeated at least twice using five wells for each condition.

### 4.4. Cell Cycle Analysis

Cell cycle analysis was performed by flow cytometry to obtain information on the mechanism of the antiproliferative effect of CCF, as previously described [51]. Initially, HeLa cells were seeded into 24-well plates at 50,000 cells/well density and incubated overnight for proper cell attachment. The cells were then treated with 2.5, 5, or 10 µM CCF and incubated for 24 h. The harvested cells were centrifuged at 1400 rpm for 5 min at room temperature. The pellets were then resuspended and stained with 10 μg/mL propidium iodide (PI), 0.1% Triton-X, 10 μg/mL RNase A, and 0.1% sodium citrate dissolved in phosphate-buffered saline (PBS, Capricorn Scientific) for 30 min in a dark place at room temperature. The measurement of the DNA content of cells was performed using a FACSCalibur flow cytometer (BD Biosciences, Franklin Lakes, NJ, USA). Cell debris and aggregates were excluded by appropriate gating. Data were analyzed using ModFit LT 3.3.11 software (Verity Software House, Topsham, ME, USA), where untreated cells were considered a control.

### 4.5. Hoechst and Propidium Iodide Fluorescent Double Staining

Cell morphology and membrane integrity changes were investigated using Hoechst 33258 and propidium iodide fluorescent double staining (HOPI). HeLa cells were seeded in 12-well plates at a 50,000 cell/well density and incubated overnight. The cells were then treated with 1 and 2 µM concentrations of the test compound and incubated for 24 h. The medium was gently removed from each well, and 1 mL of fresh medium containing the formulated staining solution (5 mg/mL Hoechst and 1 mg/mL PI) was added and incubated for 90 min in a dark place at room temperature. Finally, at least ten pictures were taken per each condition using a Nikon Eclipse TS100 fluorescence microscope (Nikon Instruments Europe, Amstelveen, The Netherlands) equipped with appropriate optical blocks for Hoechst 33258 (excitation, 360/40 nm bandpass filter; emission, 460/50 nm bandpass filter; and 400 nm dichromatic mirror) and propidium iodide (excitation, 500/20 nm bandpass filter; emission, 520 nm longpass filter; and 515 nm dichromatic mirror). The images obtained using a CCD camera (QImaging MicroPublisher Color RTV5.0, Teledyne Photometrics, Tucson, AZ, USA) were analyzed with ImageJ 1.54d software (National Institute of Health, Bethesda, MD, USA), and the ratio of intact, necrotic, and apoptotic cell populations was determined for each condition.

### 4.6. Cell Migration Assay

The migration ability of cells after treatment was measured using a wound-healing assay. HeLa cells were seeded in a 24-well plate at a density of 50,000 cells/well and maintained in a serum-reduced medium (2% FBS), and implanted in special silicone inserts (Ibidi GmbH, Gräfelfing, Germany) as an in vitro model of wound induction. After overnight incubation, the inserts were gently removed and the cells were washed with PBS, treated with the desired concentrations (1 and 2 µM), and incubated for 24 and 48 h, respectively. The percentage of wound closure was determined for each sample after 0, 24, and 48 h after exposure using ImageJ 1.54d software (National Institute of Health, Bethesda, MD, USA).

### 4.7. Cell Invasion Assay

Boyden chambers (Corning, Corning, NY, USA), representing in vitro models of the extracellular environment, were used to determine the anti-invasive effects of HeLa cells upon treatment with the desired compound. While the lower parts of the chambers were supplemented with 10% FBS medium as a chemo-attractant, cells were supplemented in serum-free medium containing the desired concentrations (1 and 2 µM). They were added to the prehydrated upper part of the chambers. After 24 h of incubation, the supernatants and non-invading cells were carefully removed with a cotton swab, and the membranes were then washed with PBS twice. Subsequently, the samples were fixed in ice-cold 96% ethanol (Reanal, Budapest, Hungary) and left for around 20 min for proper fixation. Samples were then stained with 1% crystal violet dye for 30 min in a dark place at room temperature. Finally, proper images (at least five per insert) were taken using a Nikon Eclipse TS100 microscope. Invading cells were counted and expressed as a percentage.

### 4.8. Antiestrogenic Activity

T47D cells modified to express an estrogen-responsive luciferase (Luc) reporter gene (T47D-KBluc, American Tissue Culture Collection, Manassas, VA, USA) were used to describe the possible antiestrogenic activity of CCF [52]. Cells were maintained in phenol red-free RPMI-1640 medium completed with 10% FBS and penicillin-streptomycin antibiotics. Cells were seeded on 96-well white flat-bottom plates (Greiner Bio-One, Mosonmagyaróvár, Hungary) at 50,000/well density and attached for 48 h. Then, a medium containing 10% charcoal dextran-treated FBS was applied for another 72 h. Finally, cells were treated with CCF in the concentration range of 0.47–60 µM in the presence of 40 pM 17β-estradiol, and fulvestrant was applied as a reference compound. After incubation for 24 h, the medium was removed, and 30 µL of One-Glo firefly luciferase reagent (Promega, Madison, WI, USA) was added to each well. The luminescence signal was determined (FLUOstar Optima, BMG Labtech, Ortenberg, Germany) after 3 min of incubation at room temperature according to the manufacturer’s protocol. Sigmoidal curves representing luciferase enzyme activity were fitted using GraphPad Prism version 5.01 (GraphPad, San Diego, CA, USA).

### 4.9. Statistical Analysis

Data from all cell-based experiments were statistically analyzed using variance analysis followed by the Dunnett post-test. Data were expressed as mean values ± standard error of the mean (SEM) using GraphPad Prism version 5.01 (GraphPad, Sandiego, CA, USA). The calculated *p*-values show statistical differences between study groups: *, **, and *** indicate *p* < 0.05, *p* < 0.01, and *p* < 0.001, respectively, compared to untreated control samples.

## 5. Conclusions

In conclusion, recently isolated from *Centrapalus pauciflorus* CCF demonstrated outstanding antiproliferative activity against the HVP-18-positive human cervical cancer cell line (HeLa) and considerable tumor selectivity against non-cancerous cells. It elicited G0/G1 cell cycle arrest, and the hypodiploid cell population cumulated. The apoptosis-inducing property of CCF was confirmed by identifying the nuclear condensation morphological apoptotic marker using fluorescent staining. It also displayed potent antiestrogen behavior, as evidenced by the luciferase reporter assay. Based on these findings, Centrapalus coumarin F may be regarded as a promising candidate and a starting structure for designing innovative anticancer compounds.

## Figures and Tables

**Table 1:** The antiproliferative properties of CCF against the applied cell lines.

Cell Line	Growth Inhibition (% ± SEM)		Calculated IC_50_ Value (μM)	Cisplatin IC_50_ (μM)
	10 μM	30 μM		
HeLa ^1^	69.48 ± 1.30	94.60 ± 0.55	2.28	12.43
SiHa ^1^	20.83 ± 1.68	40.84 ± 1.49	n.d. ^2^	4.29
C33A	37.32 ± 1.89	65.91 ± 0.67	15.69	
MCF7 ^1^	57.26 ± 1.05	69.71 ± 1.01	6.59	5.78
MDA-MB-231 ^1^	<20 ^3^	26.23 ± 1.38	n.d.	10.17
T47D	35.65 ± 1.53	53.06 ± 0.88	25.55	
A2780 ^1^	37.22 ± 3.02	67.96 ± 2.01	15.41	1.30
UPCI-SCC-131	24.50 ± 3.21	38.01 ± 2.87	n.d.	
UPCI-SCC-154	32.61 ± 2.20	52.21 ± 1.14	36.37	
U-87	22.25 ± 3.02	62.60 ± 1.96	21.54	
NIH/3T3	28.59 ± 2.18	51.38 ± 1.46	28.52	

^1^ Data from reference [18]. Cisplatin was included as a reference compound. ^2^ IC_50_ values are not determined when less than 50% growth inhibition was obtained at 30 μM. ^3^ An inhibition value less than 20% is considered negligible and is not given numerically.

## Data Availability

The data presented in this study are available upon request from the corresponding author (I.Z.).

## Abbreviations

The following abbreviations are used in this manuscript:

CCF	Centrapalus coumarin F
DMSO	Dimethylsulfoxide
FBS	Fetal bovine serum
HO	Hoechst 33258
HPV	Human papillomavirus
MTT	3-(4,5-dimethylthiazol-2-yl)-2,5-diphenyltetrazolium bromide
PBS	Phosphate-buffered saline
PI	Propidium iodide
SEM	Standard error of the mean
